# A Small Non-Coding RNA Mediates Transcript Stability and Expression of Cytochrome bd Ubiquinol Oxidase Subunit I in *Rickettsia conorii*

**DOI:** 10.3390/ijms24044008

**Published:** 2023-02-16

**Authors:** Hema P. Narra, Jessica Alsing, Abha Sahni, Michelle Montini, Yasim Zafar, Sanjeev K. Sahni

**Affiliations:** Department of Pathology, Institute for Human Infections and Immunity, University of Texas Medical Branch, Galveston, TX 77555, USA

**Keywords:** *Rickettsia conorii*, non-coding RNA, cytochrome bd oxidases, CydAB, small RNA mediated gene regulation, host–pathogen interaction, pXG plasmids

## Abstract

Small regulatory RNAs (sRNAs) are now widely recognized for their role in the post-transcriptional regulation of bacterial virulence and growth. We have previously demonstrated the biogenesis and differential expression of several sRNAs in *Rickettsia conorii* during interactions with the human host and arthropod vector, as well as the in vitro binding of *Rickettsia conorii* sRNA *Rc*_sR42 to bicistronic cytochrome bd ubiquinol oxidase subunits I and II (*cydAB*) mRNA. However, the mechanism of regulation and the effect of sRNA binding on the stability of the *cydAB* bicistronic transcript and the expression of the *cydA* and *cydB* genes are still unknown. In this study, we determined the expression dynamics of *Rc*_sR42 and its cognate target genes, *cydA* and *cydB*, in mouse lung and brain tissues during *R. conorii* infection in vivo and employed fluorescent and reporter assays to decode the role of sRNA in regulating cognate gene transcripts. Quantitative RT-PCR revealed significant changes in the expression of sRNA and its cognate target gene transcripts during *R. conorii* infection in vivo, and a greater abundance of these transcripts was observed in the lungs compared to brain tissue. Interestingly, while *Rc*_sR42 and *cydA* exhibited similar patterns of change in their expression, indicating the influence of sRNA on the mRNA target, the expression of *cydB* was independent of sRNA expression. Further, we constructed reporter plasmids of sRNA and *cydAB* bicistronic mRNA to decipher the role of sRNA on CydA and CydB expression. We observed increased expression of CydA in the presence of sRNA but detected no change in CydB expression in the presence or absence of sRNA. In sum, our results demonstrate that the binding of *Rc*_sR42 is required for the regulation of *cydA* but not *cydB*. Further studies on understanding the influence of this interaction on the mammalian host and tick vector during *R. conorii* infection are in progress.

## 1. Introduction

*Rickettsia conorii*, the etiologic agent of Mediterranean spotted fever (MSF), is an obligately intracellular α-proteobacterium primarily transmitted to humans by the pantropical dog tick *Rhipicephalus sanguineus* [1,2]. Upon infection of the human host via a tick bite, the bacteria exhibit tropism for the microvascular endothelium lining the blood vessels, and infected macrophages are known to play a role in further dissemination throughout the body [1,3,4]. Clinical symptoms of MSF include fever, headache, rashes, vomiting, diarrhea, and tache noire at the site of the tick bite, which can result in significant mortality [5,6,7]. These virulence-related manifestations in accidental human hosts are in sharp contrast to a relatively dormant phenotype observed in infected ticks allowing for transovarial/transstadial transmission to progeny, which supports rickettsial existence and maintenance in nature within the arthropod vector. It is now established that posttranscriptional regulation of the bacterial coding transcriptome by small non-coding RNAs (sRNAs) plays a pivotal role in regulating stress responses, virulence gene expression, and adapting to different host environments [8,9].

The discovery, prevalence, and diversity of the non-coding transcriptome in bacteria have witnessed robust progress in recent years, and a myriad of sRNAs involved in gene regulation have been identified in several bacterial species [8,10,11]. While the trans-acting sRNAs originating from intergenic regions are known to regulate multiple target genes through complementary base pairing involving a stretch of 5–7 nucleotides, the cis-acting sRNAs, originating from the non-coding strand of an open reading frame, are shown to regulate the expression of the complementary cognate gene [9,12]. Several *E. coli* sRNAs, including AcrZ, GcvB, MicA, and CyaR, are shown to regulate more than 100 target genes involved in different physiological pathways [13,14]. Most recently, dual-function sRNAs, which are chimeric transcripts originating from the intergenic regions, have been identified and are shown to regulate the target genes by direct base pairing and by encoding for a small protein that can also regulate the expression of the target protein. For instance, the *Vibrio cholerae* chimeric transcript VcdRP expresses a non-coding sRNA, VcdR, involved in reducing cholera toxin production via direct base pairing with target genes and encoding for a 29 amino acid protein, VcdP, which binds to citrate synthase (GltA) resulting in increased enzymatic activity. Both non-coding VcdR sRNA and coding VcdP protein in combination regulate carbon transport and metabolism during host–pathogen interactions [15]. Similarly, *E. coli* sRNA SgrS also encodes for a small protein, SgrT, and both SgrS and SgrT are known to down-regulate the target glucose permease PtsG via inhibition of mRNA translation and protein function, respectively [16,17].

Despite dense genomes with reduced intergenic regions, obligately intracellular bacteria also encode several sRNAs involved in the regulation of virulence, persistence, and growth [18,19,20]. The *Coxiella burnetti* trans-acting sRNA CbsR12 is not only shown to regulate genes involved in pyrimidine biosynthesis and the methionine cycle but is also required for the expansion of *Coxiella*-containing vacuoles and downregulation of the cvpD effector protein transcripts during host cell infection [21]. An average of 34 anti-sense sRNAs originating from the lagging strand of the genes involved in amino acid biosynthesis and nucleotide synthesis pathways are identified in *Carsonella*, a nutritional endosymbiont of psyllids [22]. Using high-throughput transcriptomic approaches, we identified the sRNAome of *Rickettsia* species belonging to both the spotted fever and typhus groups, determined the sRNA transcription start sites, confirmed their expression via Northern blotting, and predicted the target genes regulated by trans-acting sRNAs [23,24,25]. Our comparative transcriptomic analysis further identified several differentially and uniquely expressed rickettsial sRNAs during host–pathogen and vector–pathogen interactions in vitro, thus suggesting the role of these non-coding transcripts in the regulation of virulence in the human host and maintenance in tick vectors, respectively [26,27]. Additionally, we employed an electrophoretic mobility shift assay (EMSA) to show the binding of *R. conorii* trans-acting sRNA *Rc*_sR42 to cytochrome bd ubiquinol oxidase subunit I and II transcripts; however, the implication of this sRNA–mRNA interaction remains elusive. In this study, we sought to determine the influence of *R. conorii* sRNA *Rc*_sR42 on the transcript stability and expression of *cydA* and *cydB* genes that are transcribed as a bicistronic messenger RNA.

## 2. Results

### 2.1. Transcriptional Changes in R. conorii Rc_sR42, cydA, and cydB during Infection of Mouse b.End3 Cells In Vitro

**Figure 1:**
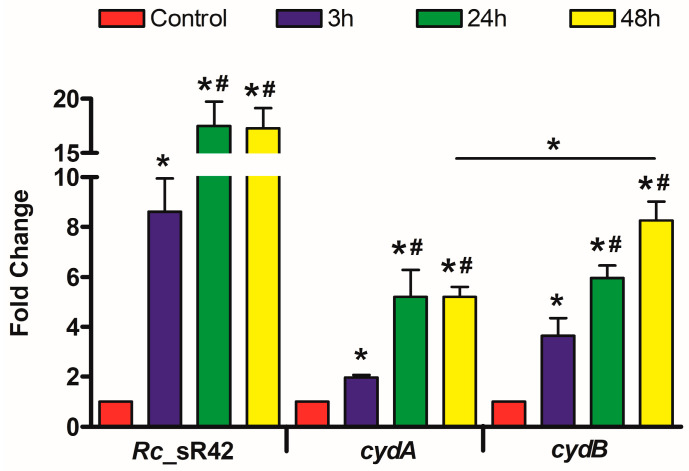
Expression profile of *Rc*_sR42 sRNA, *cydA*, and *cydB* coding transcripts in *R. conorii* during the infection of mouse endothelial (b.End3) cells in vitro. Significant upregulation of both sRNA and coding gene (*cydA* and *cydB*) transcripts was observed at 3, 24, and 48 h post-infection when compared to the control. Data are presented as mean ± SEM from three independent biological replicates. **#** Denotes significant changes (*p* < 0.05) observed between 3 h vs. 24 h and 3 h vs. 48 h. * and/or # *p* < 0.05.

Our initial studies focused on the transcriptional profiling of *R. conorii* sRNAs, and we identified *Rc*_sR42 as one of the highly abundant and differentially expressed sRNAs during host–pathogen and vector–pathogen interactions in vitro. We further confirmed the independent expression of *Rc*_sR42 and identified *cydAB* as its cognate target gene by performing electrophoretic mobility shift assays [24]. However, the expression dynamics of *cydA* and *cydB* transcripts during expressional changes of sRNA *Rc*_sR42 are currently unknown. Hence, we performed quantitative RT-PCR to measure concurrent changes in the expression of sRNA and its cognate gene transcripts in *R. conorii* during the infection of mouse endothelial cells (b.End3) in vitro. All transcripts (*Rc*_sR42, *cydA,* and *cydB*) were significantly upregulated at 3, 24, and 48 h post-infection. sRNA was ~8-fold higher at 3 h and ~17-fold upregulated at 24 and 48 h post-infection compared to the control (Figure 1). Interestingly, while *cydA* was ~2-fold higher at 3 h and ~5.1-fold upregulated at 24 and 48 h, *cydB* was ~3.6-, 5.9-, and 8.2-fold upregulated at 3, 24, and 48 h post-infection, respectively (Figure 1). A significant change in the level of expression of *cydA* and *cydB* transcripts was observed at 48 h post-infection (Figure 1). Additionally, the changes in sRNA *Rc*_sR42 expression resulted in similar levels of transcriptional changes in *cydA* but not *cydB*, as observed at 24 and 48 h post-infection, thus indicating a potential influence of sRNA on *cydA* transcript levels.

### 2.2. Expression Profile of Rc_sR42, cydA, and cydB in Lung and Brain Tissues of Mice during R. conorii Infection In Vivo

**Figure 2:**
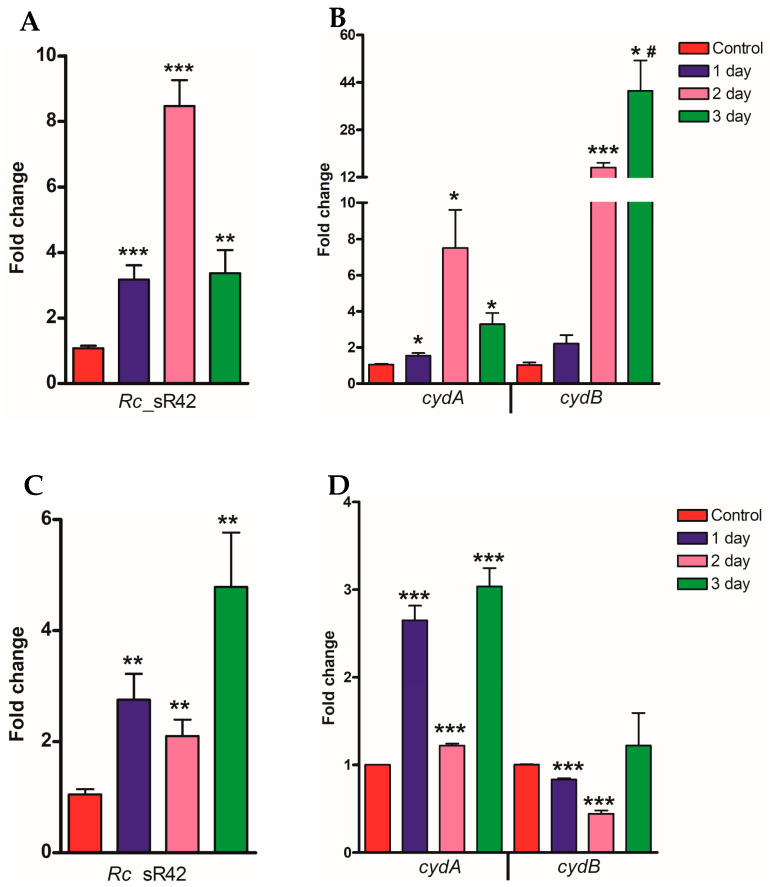
Expression profile of *Rc*_sR42 sRNA (**A**,**C**), *cydA* and *cydB* coding transcripts (**B**,**D**) in *R. conorii* during the infection of mouse lung (**A**,**B**) and brain (**C**,**D**) tissues in vivo. Significant changes in the expression of both coding (*cydA* and *cydB*) and non-coding (*Rc*_sR42) transcripts were observed at days 1–3 post-infection compared to the control. # Denotes significant changes (*p* < 0.05) observed between 3 h vs. 24 h and 3 h vs. 48 h. * *p* < 0.05, ** *p* < 0.005, *** *p* < 0.001.

To determine the changes in the expression of sRNA (*Rc*_sR42) and its cognate bicistronic target gene (*cydAB*) in vivo, we infected C3H/HeN mice with 2.25 × 10^5^ pfu of viable *R. conorii* per mouse and harvested the lungs and brain at 4, 24, 48, and 72 h post-infection. The samples collected at 4 h post-infection served as the baseline control. The expression of sRNA and *cydAB* transcripts was significantly higher in *R. conorii*-infected lung tissue compared to the brain. For instance, the expression of *Rc*_sR42 was ~8-fold and ~2-fold upregulated in the lungs and brain, respectively, at 2 days p.i. (Figure 2A,C). At 3 days p.i., *cydB* was significantly upregulated (~43-fold) in *R. conorii* during the infection of lung tissues, while no difference in its expression level was seen during the infection of brain tissue (Figure 2B,D). As observed in vitro, a positive correlation in the expression dynamics of *Rc*_sR42 and *cydA* was observed in both lung and brain tissues, while no correlation was observed between the sRNA and the *cydB* transcript (Figure 2).

### 2.3. Generation of Plasmid Constructs for Expression of Small RNAs and Their Cognate Target Gene Seed Regions

**Figure 3:**
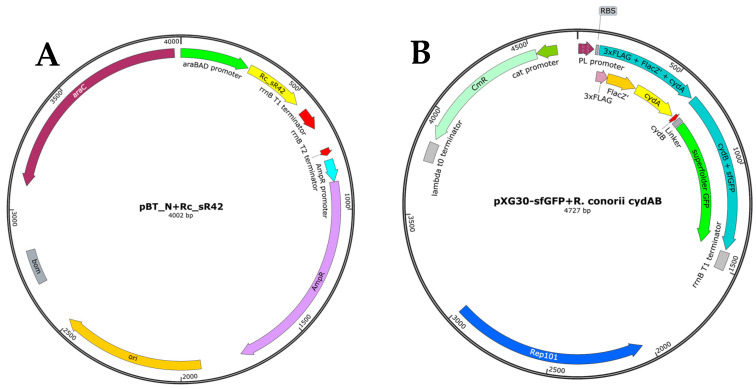
Plasmids constructed for the expression of *R. conorii* sRNA *Rc*_sR42 and partial *cydAB* bicistronic mRNA. (**A**) Plasmid map showing cloning of the sRNA downstream of an inducible arabinose promoter and upstream of a strong rrnB T1 terminator. (**B**) pXG30-sfGFP bicistronic plasmid construct expressing last 87 amino acids of *cydA* tagged to ‘FLAG-LacZ’ at the 3′ end, and first 7 amino acids of *cydB* tagged to GFP at the 5′ end of the coding sequence.

We generated plasmid constructs to decipher the role of *R. conorii* sRNA *Rc*_sR42 in *cydAB* bicistronic transcript stabilization and to determine the impact of sRNA on CydA and CydB protein expression in vitro. A full-length sRNA (*Rc*_sR42) amplified from the *R. conorii* genome was cloned in between a strong inducible arabinose promoter and rrnB1 transcriptional terminator (Figure 3A). The expression of *Rc*_sR42 during arabinose induction was verified via qRT-PCR (File S2). We employed a well-characterized expression plasmid, pXG-30sf carrying FLAG-LacZ and superfolder GFP [28], to clone the partial *cydAB* bicistronic gene. The partial *cydAB* coding region encompassing the last 87 amino acids of CydA and containing the sRNA binding seed region, intergenic bases, and the first 7 amino acids for CydB were cloned in-frame to create a single “FLAG-LacZ-*cydA*-intergenic region-*cydB*-GFP” coding frame construct under the control of a tetracycline promoter (Figure 3B). The plasmids were transformed into *E. coli* TOP10 F’ competent cells for functional characterization of the role of sRNA.

### 2.4. The R. conorii sRNA Rc_sR42 Is Required for Transcript Stabilization and Expression of cydA

**Figure 4:**
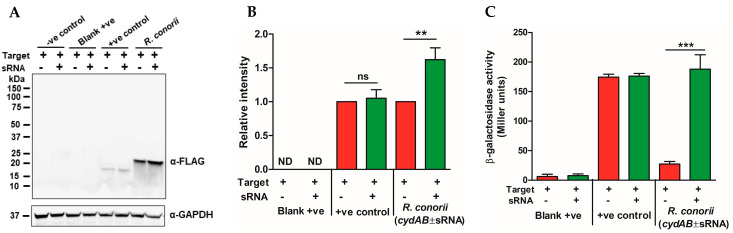
*R. conorii* sRNA *Rc*_sR42 is required for transcript stabilization and expression of *cydA*. The plasmid construct carrying the translational fusion of the seed region of *cydAB* mRNA was expressed in the presence/absence of *Rc*_sR42. The expression of FLAG-LacZ-*cydA* was measured via Western blotting and β-galactosidase assay. (**A**) Western blot image showing the expression of the FLAG fusion protein as a measure of translational fusion and GAPDH as the loading control. (**B**) Quantification of FLAG expression from at least three independent experiments presented as mean ± SEM. (**C**) Quantification of the expression of the ‘FLAG-LacZ-CydA’ fusion protein via β-galactosidase assay (n ≥ 3). The absorbance observed in negative control served as a baseline. ns = not significant, ** *p* < 0.01, *** *p* < 0.001. The pXG0 plasmid containing luciferase served as a blank negative; pXG1 plasmid carrying GFP directly under the control of aTc promoter served as blank positive control; and *E. coli glmUS*, known to be regulated by glmZ sRNA served as positive control.

To test the impact of *Rc*_sR42 on the expression of the CydA protein, we transformed the plasmid constructs reported above (Figure 3A,B) into *E. coli* TOP10 F’ cells and induced the expression of sRNA and the “FLAG-LacZ-*cydA*-intergenic region-*cydB*-GFP” bicistronic construct with arabinose and aTc, respectively, as described in methods. As “FLAG-LacZ-CydA” is expressed as a single recombinant protein, we tested the FLAG expression through Western blot and LacZ activity via the β-galactosidase assay, as a direct measure for determining the impact of sRNA on *cydA* transcript stability and translation. Interestingly, the induction of sRNA significantly increased CydA protein expression when compared to the control containing no sRNA expression (Figure 4A,B). The *E. coli* GlmZ sRNA is known to increase the stabilization and expression of GlmS with no impact on GlmU within the bicistronic GlmUS transcript [28,29]. Accordingly, we observed similar levels of GlmU expression both in the presence and absence of GlmZ sRNA. The quantification of the expression of ‘FLAG-LacZ-CydA’ fusion protein via β-galactosidase assay resulted in a similar level of increase in the expression of CydA protein in the presence of sRNA (Figure 4C), thus showing a positive influence of sRNA on the transcript stability and expression of CydA. 

### 2.5. The R. conorii sRNA Rc_sR42 Is Not Involved in the Regulation of cydB

**Figure 5:**
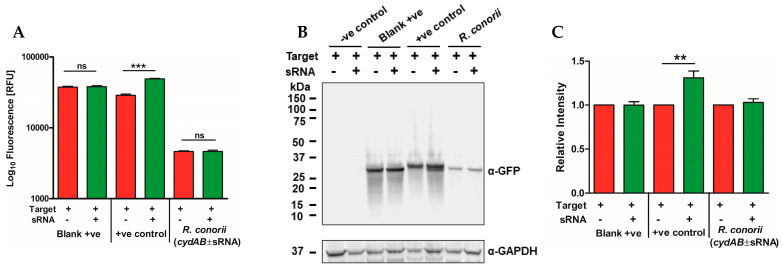
Expression of *R. conorii cydB* in the presence or absence of sRNA *Rc*_sR42. The bicistronic *cydAB* transcript containing the sRNA binding seed region was cloned into a pXG-30sf plasmid and expressed in *E. coli* in the presence or absence of a plasmid expressing sRNA *Rc*_sR42. The proteins “FLAG-LacZ-*CydA*” and “CydB-GFP” (in-frame) were induced as described in the Methods section. The expression of GFP was measured to determine the impact of *Rc*_sR42 on the expression and stability of *cydB* transcript. (**A**) Fluorescence (green) intensities of live cultures measured using a SpectraMax iD plate reader. The readings from negative control were used as a baseline, and data are shown as relative fluorescence units (RFU). Data are presented as mean ± SEM (n = 3). (**B**) Representative Western blot image showing the expression of CydB-GFP (probed with α-GFP antibody) in the presence or absence of sRNA expression. GAPDH served as the sample loading control. (**C**) Quantification of GFP expression (band intensity) from at least three independent Western blot experiments. Data are presented as mean ± SEM. ns = not significant, ** *p* < 0.01, *** *p* < 0.001. The pXG1 plasmid carrying GFP directly under the control of aTc promoter served as blank positive control, while *E. coli glmUS*, known to be regulated by glmZ sRNA served as positive control.

As *cydAB* is expressed as a bicistronic transcript in *R. conorii*, we tested if the expression of *Rc*_sR42 influences the expression of *cydB*. The expression of the *cydB*-GFP fusion protein was measured through fluorescence as well as Western blotting in the presence and absence of sRNA expression. Interestingly, sRNA expression did not influence *cydB* expression, and similar levels of GFP fluorescence intensity were observed in both the presence and absence of sRNA expression (Figure 5A). As expected, the *E. coli* sRNA GlmZ had a positive effect on the expression of GlmS and was involved in transcript stabilization and production of GlmS protein (Figure 5A). These results were further confirmed via Western blot analysis using α-GFP antibodies (Figure 5B,C).

## 3. Discussion

Small noncoding RNAs are essential for posttranscriptional regulation of gene expression in all organisms, including bacteria, and are known to play a role in virulence, adaptation to stress, starvation, environmental stimuli, and resistance to antibiotics [9,11,12]. In this study, we have determined the expression dynamics of a trans-acting *R. conorii* sRNA, *Rc*_sR42, and its cognate target genes and established a role for the sRNA in transcript stabilization and expression of cytochrome bd ubiquinol oxidase subunit I (*cydA*) using reporter assays. As dynamic changes in the expression of sRNA can impact the expression of true cognate target genes, we saw a direct correlation of transcript level changes between *Rc*_sR42 and *cydA* transcript in cell culture-based systems in vitro and animal models of experimental infection in vivo. Using reporter assays, we have further validated that the binding of *Rc*_sR42 at the 3′ end of the *cydA* transcript within the bicistronic cytochrome bd ubiquinol oxidase subunits I and II (*cydAB*) transcript results in transcript stabilization and expression of its cognate target gene (*cydA*).

Cytochrome bd is a transmembrane terminal respiratory oxidoreductase that primarily reduces oxygen into the water while producing a proton motive force for ATP synthesis [30]. The cytochrome bd is composed of two main subunits, namely CydA (57 kDa) and CydB (43 kDa), that are known to contain three active redox cofactors (heme *b_558_*, heme *b_595_*, and heme *d*) and the Q-loop (in CydA) for quinol binding and oxidation [31]. Recently a smaller subunit (*cydX*) encoding for a 4 kDa protein involved in the stabilization of the hemes was identified in several bacterial species belonging to α, β, and ɤ-proteobacteria [32,33]. Several factors, including hypoxia, inhibitors of cell wall biosynthesis and oxidative phosphorylation, antibacterial compounds, and reactive oxygen and nitrogen species (ROS and RNS), are known to stimulate the expression of *cydAB* genes [34]. Evidence suggests that the deletion of cytochrome bd renders bacteria sensitive to hydrogen peroxide-induced oxidative stress, as exhibited by *E. coli cydAB* deletion mutants [35]. Further, among gut microbes, including *E. coli*, the cytochrome bdI and bdII oxidases were active and insensitive to hydrogen sulfides (H_2_S) produced in the intestine, while the activity of heme–copper oxidases (cytochrome *bo_3_*) was completely inhibited by H_2_S [36]. Owing to these unique attributes rendered by cytochrome bd oxidases, it is likely that the genomes of several bacterial pathogens, such as *Salmonella*, *Shigella*, *Mycobacterium*, *Listeria*, *Brucella*, and *Klebsiella*, harbor and encode *cydAB*. Consistent with these observations, we have also identified that all *Rickettsia* species belonging to spotted fever, typhus, transitional, and ancestral groups also encode for bicistronic *cydAB* genes in their genomes, implicating that these genes likely provide protection to the bacterial cell and that their expression is critical for rickettsial survival and persistence during in vitro and in vivo infection. However, we did not identify orthologs of *cydX* in *Rickettsia* species, possibly due to sequence divergence, as exemplified by the lack of homology between *E. coli cydX* and *Geobacillus thermodenitrificans cydS* genes, despite both genes coding the smaller subunit of the protein complex [31].

As obligately intracellular pathogens, *Rickettsia* species establish a niche in the nutrient-rich cytosol of host cells within 12–15 min of infection [37]. Upon internalization, the bacteria immediately exhibit metabolic activity, consume oxygen, and release CO_2_, and O_2_ consumption was found to be directly proportional to the number of viable bacteria present in the cytosol [38]. This colonization and replication of *Rickettsia* inside the host cells result in hypoxia, hypercapnia, nutrient depletion, and the production of ROS and RNS during the later stages of the growth. The expression of *cydAB* is known to make bacterial cells resistant to stress and hypoxia conditions, promote respiration and growth in adverse conditions, and aid in ATP synthesis during nutrient-limiting conditions. After entry into the host cell, rickettsiae import ATP from the host cytosol via ATP/ADP translocases, and, upon depletion, the bacteria utilize aerobic respiration involving CydAB for the synthesis of their ATP [39,40]. Additionally, cytochrome bd oxidases (CydAB) are shown to exhibit a higher affinity (>1000 fold) for oxygen when compared to other bacterial or human cytochromes and aid in scavenging oxygen in hypoxic cytosol during intracellular infection [41]. A 50-fold upregulation of *Mycobacterium cydAB* was observed in hypoxia and extreme carbon-limiting conditions [42]. In uropathogenic *E. coli* (UPEC), the deletion of *cydAB* resulted in reduced intracellular replication, oxygen consumption, nitric oxide tolerance, and extracellular acidification rates. Further, *cydAB* was shown to be involved in antagonizing pro-apoptotic factors and rewiring of host cell metabolism during UPEC infection of urothelial cells, and the amino acid lysine at position 252 in CydA was shown to be a key residue for bacterial respiration required for intracellular survival [43]. Consistent with these observations, we observed an increased expression of both *cydA* and *cydB* transcripts at 24 and 48 h, when compared to 3h post-infection (Figure 1). Additionally, both transcripts were significantly upregulated in *R. conorii* during the infection of lung and brain tissues in vivo (Figure 2). Interestingly, the transcriptional changes observed for *R. conorii cydA* were directly proportional to the changes observed in *Rc*_sR42, thus indicating that both sRNA and *cydA* collectively may contribute to rickettsial survival, ATP synthesis, and replication during infection via sRNA-mediated stabilization of the *cydA* transcript (Figure 4).

Most bacterial trans-acting sRNAs are known to bind in the promoter region of the transcript, resulting in either translation initiation or inhibition depending on the availability of the ribosomal binding site for translation [8,11]. However, a few sRNAs are known to bind in the intercistronic regions. For instance, the *E. coli* Spot 42 sRNA is shown to bind at the intercistronic region of *galT-galK* within the *galETKM* polycistronic mRNA, resulting in transcript cleavage and the generation of stable *galET* transcripts [44]. In this study, we have shown that the binding of *R. conorii* sRNA, *Rc*_sR42, at the 3′ end of *cydA* within the bicistronic *cydAB* transcripts results in transcript stabilization and expression of *cydA* (Figure 4). Thus, it is likely that the binding of sRNA within the polycistronic transcript results in the production of relatively stable upstream transcripts. Interestingly, we did not observe any correlation between the transcriptional changes in *cydB* and *Rc*_sR42 (Figure 1 and Figure 2). Additionally, we have previously confirmed the bicistronic expression of *R. conorii cydAB* during host endothelial cell infection in vitro (File S1). Further in-depth analysis of the *R. conorii* genome resulted in the identification of a putative promoter region within the *cydAB* operon, which is located 150 bases upstream of the *cydB* start codon (File S1). Consistent with this observation, we have recently identified primary transcripts mapping to >900 intragenic transcription start sites in the *R. conorii* genome [27]. Hence, it is likely that, apart from being expressed as a part of a bicistronic transcript, *cydB* is also expressed as an independent transcript, resulting in differences in expression between *cydA* and *cydB*, despite being organized as a bicistronic operon. However, studies aimed at functional characterization of the identified putative promoter region will provide further insight into the expression dynamics of *cydAB* in vitro and in vivo.

Although the generation of sRNA deletion mutants offers a direct approach for the identification of bonafide target genes, the generation of mutant strains in obligately intracellular pathogens including *Rickettsia* species remains a challenge largely due to the lack of appropriate genetic tools and protocols and the time-consuming, laborious procedures required for the purification and expansion of clonal populations [45]. Hence, we utilized a well-established and extensively used pXG plasmid-based reporter system to decipher the influence of *R. conorii* sRNA on its cognate target genes [28,46,47]. This system allows the measurement of the up- or down-regulation of the target gene through multiple ways-fluorescence, Western blotting, and/or biochemical assays. In this study, we employed two independent assays for each target gene and determined the influence of sRNA on *cydA* via Western blotting and the β-galactosidase assay and *cydB* via GFP fluorescence and Western blotting. Both assays provide conclusive evidence of the role of *Rc*_sR42 in the regulation of *cydA* but not *cydB* transcripts. Consistent with our observations, *E. coli* RyhB sRNA was shown to directly regulate *cydAB* during iron-limiting conditions [48].

In summary, we herein showed for the first time that the expression of *R. conorii* trans-acting sRNA *Rc*_sR42 is involved in transcript stabilization and expression of cytochrome bd ubiquinol oxidase subunit I (*cydA*), which is involved in bacterial respiration and in conferring resistance to oxidative stress conditions during survival and replication in the host cytosol. We have further shown that the transcriptional changes observed in *cydA* are tightly correlated and directly proportional to the changes in sRNA transcript levels. Ongoing research including identification and mutational analysis of the key residues in the sRNA–mRNA seed region will provide better insights into the mechanisms of base pairing to further delineate the influence of this interaction during host–pathogen and vector–pathogen interactions.

## 4. Materials and Methods

### 4.1. Bacterial Strains

*Rickettsia conorii* strain Malish7 was grown in Vero cells, purified, and stored at −80 °C, as previously described [49,50]. Briefly, monolayers of Vero cells were infected with a seed stock of *R. conorii* (MOI = 1) and grown at 35 °C, 5% CO_2_ in Dulbecco’s Modified Eagle medium (Coring, Manassas, VA, USA) containing 2% fetal bovine serum (FBS) (HyClone, Logan, UT, USA) until ~10–15% of the monolayer was lysed or detached (~4–5 days post-infection) from the surface. The Vero cells containing the bacteria were harvested, and *R. conorii* was purified by differential centrifugation. The purified rickettsial stock was suspended in K36 buffer (100 mM potassium chloride, 15 mM sodium chloride, 50 mM potassium phosphate buffer [pH 7.0]), aliquoted in <500 µL, and stored at −80 °C. The homogeneity of purified rickettsial stock was assessed using Diff-Quik staining (Siemens, Newark, DE, USA) and quantified via citrate synthase (*gltA*)-based qPCR and plaque assays, as described [24,49].

*E. coli* strains DH5α and TOP10 F’ (ThermoFisher Scientific, Waltham, MA, USA) were grown in LB medium at 37 °C unless otherwise stated. All *E. coli* stocks were stored in 15% glycerol at −80 °C.

### 4.2. Generation of Plasmid Constructs

The plasmid backbone for cloning small RNA was generated by modifying the pBAD/Thio-TOPO vector (ThermoFisher Scientific, Waltham, MA, USA). Briefly, inverse PCR using primer pair pBT-F and pBT-R and pBAD/Thio plasmid as template DNA was performed to remove the HP-thioredoxin-EK site, V5 epitope, and 6X His-tag from the vector and to insert *Eco*RI, *Kpn*I, and *Xho*I restriction sites into the modified plasmid. The amplified linear PCR product was cleaned using SV gel and PCR purification kit (Promega, Madison, WI, USA), digested with *Kpn*I (New England Biolabs, Ipswich, MA, USA), and circularized using T4 DNA ligase (New England Biolabs, Ipswich, MA, USA). The modified plasmid containing an arabinose inducible promoter, unique restriction sites listed above, and strong rrnB terminator was verified via Sanger sequencing and named pBT_N. Full-length sRNA, either amplified from *R. conorii* (*Rc*_sR42 sRNA) or *E. coli* (glmZ sRNA) genomic DNA, was cloned into pBT_N plasmid backbone using the unique restriction sites, thus resulting in an sRNA expression plasmid under the tight control of an inducible arabinose promoter and strong transcription terminator. The plasmid containing the sRNA binding target gene seed region was constructed by cloning the target gene amplicon in-frame into pXG30-superfolder GFP (sfGFP) plasmid [28], which mimics an intra-operonic target arrangement (gift from Prof. Jörg Vogel, Helmholtz Centre for Infection Research, Germany). The anhydrotetracycline (aTc) inducible pXG30-sfGFP was specifically chosen for these studies as the binding region of sRNA *Rc*_sR42 is known to be present at the 3′ end of the *cydA* gene, which is expressed as a bicistronic *cydAB* operon in *R. conorii* [24,28]. Briefly, the *R. conorii cydAB* operonic gene fragment encoding for the last 87 amino acids of *cydA* and containing the predicted seed region, intergenic bases, and the first 7 amino acids for the *cydB* gene was amplified using the primer pair cydAB-30F and cydAB-30R and cloned in-frame into the linearized pXG30-sfGFP plasmid using *Nsi*I and *Nhe*I restriction sites. The resulting *cydAB* intra-operonic plasmid had FLAG and LacZ tagged to *cydA* at its 5′ end and GFP tagged to *cydB* at its 3′ end, allowing assessment of sRNA-mediated regulation of both the genes. The *E. coli glmUS*, known to be regulated by glmZ sRNA, was cloned into pXG30-sfGFP plasmid as described earlier and served as the positive control [29]. The pXG1 plasmid carrying GFP directly under the control of aTc promoter served as blank positive control, while the pXG0 plasmid containing luciferase served as a blank negative control in these studies. All primers used for the generation of plasmid constructs are listed in Table S1 and plasmid maps are shown in Figure 3.

### 4.3. Induction of Bacterial Cultures

To study the *R. conorii* sRNA-mediated regulation of the *cydAB* transcript, we generated *E. coli* TOP10 F’ strains carrying plasmids coding for sRNA (pBT_N backbone) and/or plasmids carrying the sRNA binding seed region of the target gene (pXG30-GFPsf backbone). The transformed *E. coli* TOP10 F’ strains (stocks) were stored in 15% glycerol at −80 °C until use. The list of bacterial strains, their respective harboring plasmids, and antibiotic resistance are provided in Table S2. The *E. coli* stocks carrying the appropriate combination of plasmids were streaked onto LB agar plates containing antibiotic(s) and incubated at 37 °C overnight. A loop of bacterial culture was inoculated in 3mL of LB Broth supplemented with appropriate antibiotic(s) and incubated overnight at 37 °C, 225 rpm. The overnight culture was diluted 1:20 using LB Broth containing antibiotic(s) and incubated at 37 °C, 225 rpm until the optical density (OD_600_) reached between 0.5 and 0.6, at which point the culture was induced by adding 0.02% arabinose for sRNA expression and/or 100 ng/mL anhydrotetracycline (aTc) for the expression of ‘FLAG-LacZ-target gene fragment-GFP’ cloned in the pXG30-GFPsf plasmid. A sample of the uninduced cultures was run in parallel in all experiments. After induction, the cultures were incubated overnight (~16 h) at 30 °C, 225 rpm. The induced and uninduced samples were used for measuring GFP fluorescence, β-galactosidase assay, and Western blot analysis as described below.

### 4.4. Measurement of GFP Fluorescence in Live Bacterial Cultures

To ensure equal growth of cultures, the samples were diluted 1:4 with water, and the OD_600_ of both the uninduced and induced samples was measured. To measure the green fluorescence intensity, the liquid bacterial cultures were diluted 1:2 in 96-well sterile cell culture plates. GFP fluorescence was measured using SpectraMax iD5 plate reader (Molecular Devices, San Jose, CA, USA) for further quantification. The autofluorescence observed in uninduced cultures served as a baseline and was subtracted from the fluorescence values observed in induced cultures.

### 4.5. β-Galactosidase Assay

The β-galactosidase activity in bacterial cultures was measured on a microplate reader as described by [51]. Briefly, bacterial cultures grown overnight after induction were diluted to obtain a cell density (*A*_600_) of ~0.25. The bacterial cells were permeabilized by adding 100 µL of culture (*A*_600_ = 0.25) to 1mL of Z buffer (60 mM Na_2_HPO_4_.7H_2_O, 40 mM NaH_2_PO_4_.H_2_O, 10 mM KCl, 1 mM MgSO_4_.7H_2_O, 50 mM β-mercaptoethanol) followed by 20 µL of freshly prepared 0.1% SDS and 40 µL of chloroform in a 1.5mL Eppendorf tube. The tubes were mixed well by vortexing, and chloroform was allowed to settle down to the bottom of the tube at room temperature. Permeabilized cells (100 µL) were transferred onto a microplate, 40 µL of O-nitrophenyl-beta-D-galactopyranoside (OPNG) was added to each well, and absorbance readings at *A*_420_, *A*_550_, and *A*_600_ were immediately taken and considered as zero time points. The plates were incubated for an hour in the dark at room temperature, 50 µL of 1M Na_2_CO_3_ was added to stop the reaction, and absorbance was determined. Data from a minimum of three independent biological replicates were analyzed and β-galactosidase activity in Miller units was calculated as described by [51].

### 4.6. Western Blotting

Bacterial cultures with equal cell density (*A_600_*) were spun down at 4000× *g* for 5 min, and the pellet was suspended in 2x SDS PAGE sample buffer. Protein lysates were separated on a denaturing polyacrylamide gel and transferred onto the nitrocellulose membrane. The membranes were blocked in 5% milk and incubated overnight with GFP and FLAG tags primary antibodies at 1:1000 dilution. A compatible horseradish peroxidase (HRP)-conjugated secondary antibody was used, followed by chemiluminescence-based detection. The blots were stripped and probed with GADPH antibody (1:2000) as a loading control to account for variation in protein loaded in individual lanes. A minimum of three independent experiments were performed for each protein, and densitometric analysis was performed using Image J software [52].

### 4.7. Cell Culture and Infection

The mouse endothelial cell line isolated from brain tissue, bEnd.3 (ATCC CRL2299), was grown in DMEM supplemented with 10% FBS at 37 °C in an atmosphere of 95% O_2_:5% CO_2_. The bEnd.3 cells were grown in 25 cm^2^ cell culture flasks to 90–95% confluence prior to infection with *R. conorii* (MOI = 5) in minimum volume (500 µL) of medium to facilitate adherence and invasion of host cells. After 15 min, additional (2.5 mL) medium was added to each flask and incubated at 37 °C, 5% CO_2_. At the end of each time point, the sample was harvested in Tri-Reagent for RNA extraction following our established protocols [50,53]. The sample harvested at 15 min post-infection served as a baseline control for all qPCR experiments. A minimum of three independent biological replicates were performed for each experiment.

### 4.8. Quantitative Real-Time PCR

Total RNA was extracted following the standard Tri-Reagent protocol. The samples were treated with DNaseI (Invitrogen, Waltham, MA, USA) to remove genomic DNA and reprecipitated using 3M sodium acetate (pH 5.2) and glycogen (Ambion, Austin, TX, USA). The purified total RNA was quantified using a MultiSkan Go spectrophotometer (ThermoFisher Scientific, Waltham, MA, USA), and first-strand synthesis of the complementary DNA (cDNA) was performed using random primers and Superscript II Taq polymerase (Invitrogen, Waltham, MA, USA) following our established protocol [27]. The expression profile of target genes at different time points was assessed through SYBR Green-based relative quantification using gene-specific primers and 16S rRNA as endogenous control. The expression of *Rc*_sR42 after induction with arabinose was verified using sRNA-specific primers and *E. coli* 16S rRNA as control (File S2). The gene expression data were analyzed via the *ΔΔC_T_* method using a sample harvested at 15 min post-infection (p.i.) as the baseline control [54]. All primer sequences used in this study are listed in Table S1.

### 4.9. Animal Studies

Six- to eight-week-old C3H/HeN mice purchased from Jackson laboratories were acclimatized in the vivarium for a minimum of three days. The mice were assigned to groups (control or treated), and baseline body weights were recorded one day before infection. Each mouse in the treated group was injected intravenously with a lethal dose (2.25 × 10^5^ pfu/mouse) of viable *R. conorii*, while control group mice received equal volume saline. Both control and treated mice were housed in an Animal Biosafety Level 3 facility following approved institutional protocol. The animals were monitored twice daily for weight loss and signs of disease. At the end of each time point (4h, 1d, 2d, and 3d p.i.), mice were euthanized, and organs (lungs and brain) were collected aseptically into RNAlater^TM^ and stored at −20 °C until RNA extraction. The tissues were homogenized using Tissue Lyser II (Qiagen, Germantown, MD, USA), and total RNA was extracted using TRI-reagent following our standard protocol. Owing to the obligately intracellular lifestyle of rickettsial pathogens, data from the tissues of mice infected for 4h served as the baseline control for quantification of the expression of the bacterial target genes on days 1–3 p.i. All protocols used in this study were reviewed and approved by the Institutional Animal Care and Use Committee (IACUC) at the University of Texas Medical Branch (UTMB), Galveston, TX, USA.

### 4.10. Statistical Analysis

A minimum of three independent biological replicates were performed for each experiment, and statistical analysis was performed using GraphPad Prism version 5.0 (GraphPad Software Inc., San Diego, CA, USA). Comparison between matched and unmatched groups was performed using a paired *t-*test and a Mann–Whitney *U-*test, respectively. The *p-*value for statistical significance was set at <0.05.

## Data Availability

All data generated during the study and supporting the reported results are included in this manuscript.

## Supplementary Materials

The following supporting information can be downloaded at: https://www.mdpi.com/article/10.3390/ijms24044008/s1.
