# Supplementary material for: A Small Non-Coding RNA Mediates Transcript Stability and Expression of Cytochrome bd Ubiquinol Oxidase Subunit I in Rickettsia conorii
Source: Int J Mol Sci. 2023 Feb 16;24(4):4008. doi: 10.3390/ijms24044008 (PMC9960880; doi:10.3390/ijms24044008)
Supplement: Supplementary file 1 [file ijms-24-04008-s001.zip › File S1.pdf]

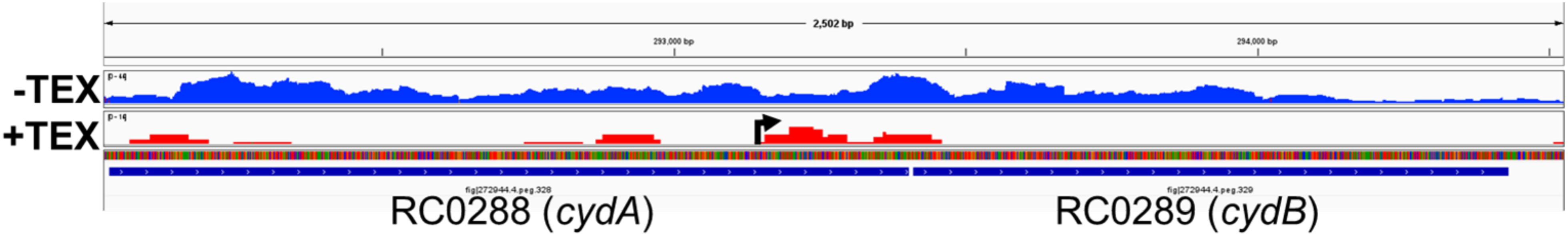

File S1: Expression of *cydAB* as bicistronic transcript in *R. conorii* during the infection of host endothelial cells in vitro [27]. **Blue:** Reads resulting from both primary and processed transcripts obtained from libraries not treated with 5′-monophosphate-dependent exonuclease (TEX). **Red:** Reads resulting only primary transcripts obtained from libraries treated with TEX. This result establish *cydAB* as a bicistronic transcript in *R. conorii*. The black arrow on +TEX reads indicate the putative transcription start site identified upstream of *cydB*.
