# Supplementary material for: A Small Non-Coding RNA Mediates Transcript Stability and Expression of Cytochrome bd Ubiquinol Oxidase Subunit I in Rickettsia conorii
Source: Int J Mol Sci. 2023 Feb 16;24(4):4008. doi: 10.3390/ijms24044008 (PMC9960880; doi:10.3390/ijms24044008)
Supplement: Supplementary file 1 [file ijms-24-04008-s001.zip › File S2.pdf]

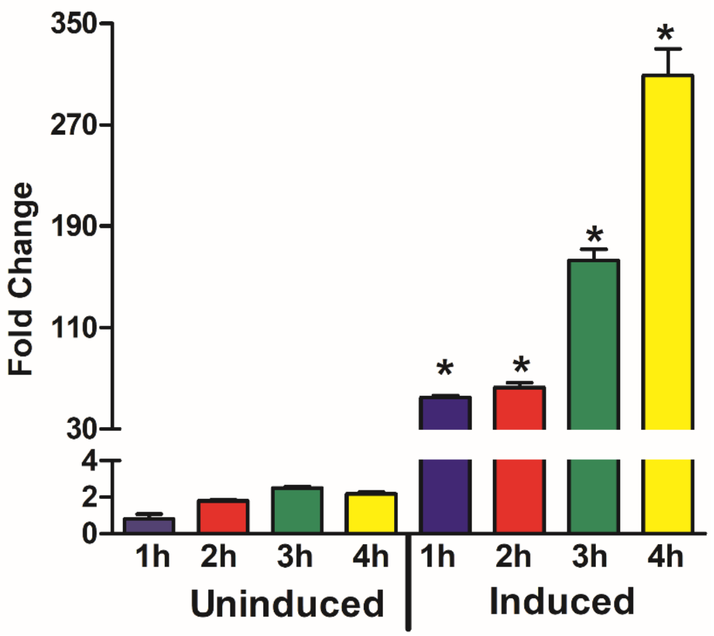

File S2: Expression profile of *R. conorii* sRNA *Rc*_sR42 in the absence or presence of arabinose. Cultures of *E. coli* Top10F′ carrying pBT_N+*Rc*_sR42 plasmid were grown at 37°C, 220 rpm until OD_{600} reached 0.5. The expression of *Rc*_sR42 was either uninduced or induced by adding 0.02% arabinose and samples were collected 1 to 4 h post OD_{600} 0.5. Total RNA from samples (1 ml of OD_{600} =1) was extracted by Tri-Reagent, DNaseI treated, and reverse transcribed as described in methods. Quantitative RT-PCR was performed using *Rc*_sR42 specific primers and 16S as endogenous control. Data was analyzed by $^{\Delta\Delta}CT$ method and presented as Mean±SEM (n≥3). A significant upregulation of *Rc*_sR42 transcript was observed post-induction with arabinose. * P<0.05
